# Medium-chain Acyl-CoA Dehydrogenase Deficiency Identified by MS/MS Newborn Screening Challenges

**DOI:** 10.34763/jmotherandchild.20252901.d-25-00025

**Published:** 2025-12-04

**Authors:** Ewa Głąb-Jabłońska, Joanna Taybert, Anna Wiśniewska, Mariola Sasin-Rokita, Mariusz Ołtarzewski, Magdalena Chełchowska

**Affiliations:** Department of Screening Tests and Metabolic Diagnostics, Institute of Mother and Child, Warsaw 01-211, Kasprzaka 17a, Poland; Department of Inborn Errors of Metabolism and Paediatrics, Institute of Mother and Child, Warsaw 01-211, Kasprzaka 17a, Poland

**Keywords:** MCAD deficiency, newborn screening, tandem mass spectrometry, C8, DBS, MS/MS, atypical acylcarnitine profile

## Abstract

**Background:**

Medium-chain acyl-CoA dehydrogenase (MCAD) deficiency is an inborn error of metabolism resulting in the absence or reduced activity of the enzyme responsible for the β-oxidation of medium-chain fatty acids. MCAD deficiency can lead to metabolic decompensation, presenting as hypoketotic hypoglycaemia, hepatic encephalopathy (Reye-like syndrome), or death regardless of the patient’s age.

**Material and methods:**

Blood samples in the national newborn screening programme were collected using 903 filter paper (dry blood spot – DBS). Routine dried blood spots from newborn screening (NBS) were analysed by flow injection and derivatised tandem mass spectrometry method (MS/MS). Positive screening cases in the MCAD deficiency profile were verified through GC/MS urine organic acid profiling and enzymatic and/or molecular testing.

**Results:**

A total of 3,806,166 newborns were screened between 2014–2024, resulting in the identification of 94 cases of MCAD deficiency. Analysis of the obtained results revealed a consistent pattern in the levels of octanoylcarnitine (C8), hexanoylcarnitine (C6), and decanoylcarnitine (C10) acylcarnitines, as well as the C8/C10 ratio across these cases. Only one case of confirmed MCAD deficiency with an atypical acylcarnitine profile was found.

**Conclusions:**

The use of tandem mass spectrometry has enabled the inclusion of MCAD deficiency in newborn screening programmes. This has facilitated early detection, diagnosis, and initiation of therapeutic interventions to prevent metabolic decompensation. We emphasize the need for repeated sampling and further testing if C8 is even slightly elevated.

## 1. Introduction

Medium-chain acyl-CoA dehydrogenase (MCAD) deficiency is caused by the absence or reduced activity of the enzyme responsible for the β-oxidation of medium-chain fatty acids. MCAD, located in the mitochondrial matrix, catalyses the first step in the oxidation of fatty acids with chain lengths ranging from C6 to C12. A deficiency of this enzyme impairs fatty acid metabolism, resulting in reduced tolerance to fasting [1]. The highest levels of C8 and the C8/C10 ratio are observed in newborns with residual MCAD enzyme activity of 0%–10%. Individuals with MCAD deficiency who have dehydrogenase activity greater than 35% are at low risk of metabolic decompensation and are often asymptomatic.

This inborn error of metabolism is caused by pathogenic variants in the *ACADM* gene (located on chromosome 1p.31), which encodes MCAD. The most common pathogenic variant in Europeans, NM_000016.5:c.985A>G (p.Lys329Glu), was associated with the highest C8 levels in neonates. Most affected individuals are homozygous for this mutation [2,6]. MCAD deficiency predisposes individuals to metabolic decompensation, typically presenting as hypoketotic hypoglycaemia or hepatic encephalopathy (Reye-like syndrome). Clinical manifestations may occur at any age, from the neonatal period to adulthood, and in severe cases, the condition can be fatal [2,3,4,5,6,7,8].

The availability of advanced technologies, particularly tandem mass spectrometry (MS/MS), has facilitated the inclusion of MCAD deficiency in newborn screening programmes in numerous countries worldwide [7]. Diagnosis is primarily based on the identification of a characteristic abnormal acylcarnitine profile in dried blood spot (DBS) samples. This approach enables early detection and diagnosis of the condition, allowing for timely preventive measures to avoid potentially life-threatening metabolic decompensation [9].

Newborns with MCAD deficiency generally do not present with clinical manifestations at birth. However, elevated concentrations of specific acylcarnitines, including C6, C8, and C10, can be detected in early infancy by MS/MS analysis and serve as diagnostic biomarkers for this disorder in NBS [1, 2, 4, 5, 10].

The final results of NBS are not always clear. Therefore, the aim of this study was to present an atypical case of MCAD deficiency detected by newborn screening with an uncharacteristic biochemical profile and to analyse factors that may influence screening results during the neonatal period.

## 2. Material and methods

Blood samples were collected from newborns at three to four days of life (as recommended). A 3.2 mm disc was punched from the sample. The analytes were extracted in 100 μL of methanol containing stable isotopes of amino acids and carnitines (internal calibrators) and derivatised with 50 μL of 3N-HCl in n-butanol at 65°C for 15 minutes [11]. Tandem mass spectrometers (API3200 and API3500, AB SCIEX, Canada) coupled to a high-pressure liquid chromatography system (Shimadzu LC20 pumps and CTC PAL autosamplers, SHIMADZU, Japan) were used for the flow injection analysis. Concentrations were calculated using in-house software and calibrators (dried blood enriched with known amounts of amino acids and carnitines). The most important biomarkers for MCAD deficiency were C8, C6, C10, and C8/C10 ratio. According to the established newborn screening protocol, elevated results for C6, C8 and C10 require either a repeated test with a new blood sample or a recall of the newborn to the clinic, depending on the C8 concentration.

MCAD deficiency was confirmed by an enzyme activity assay in lymphocytes based on the oxidation of octanoyl-CoA and the reduction of ferrocenium hexafluorophosphate. Enzyme activity was measured using high-pressure liquid chromatography (HPLC) Shimadzu with UV detector.

**Figure 1.**
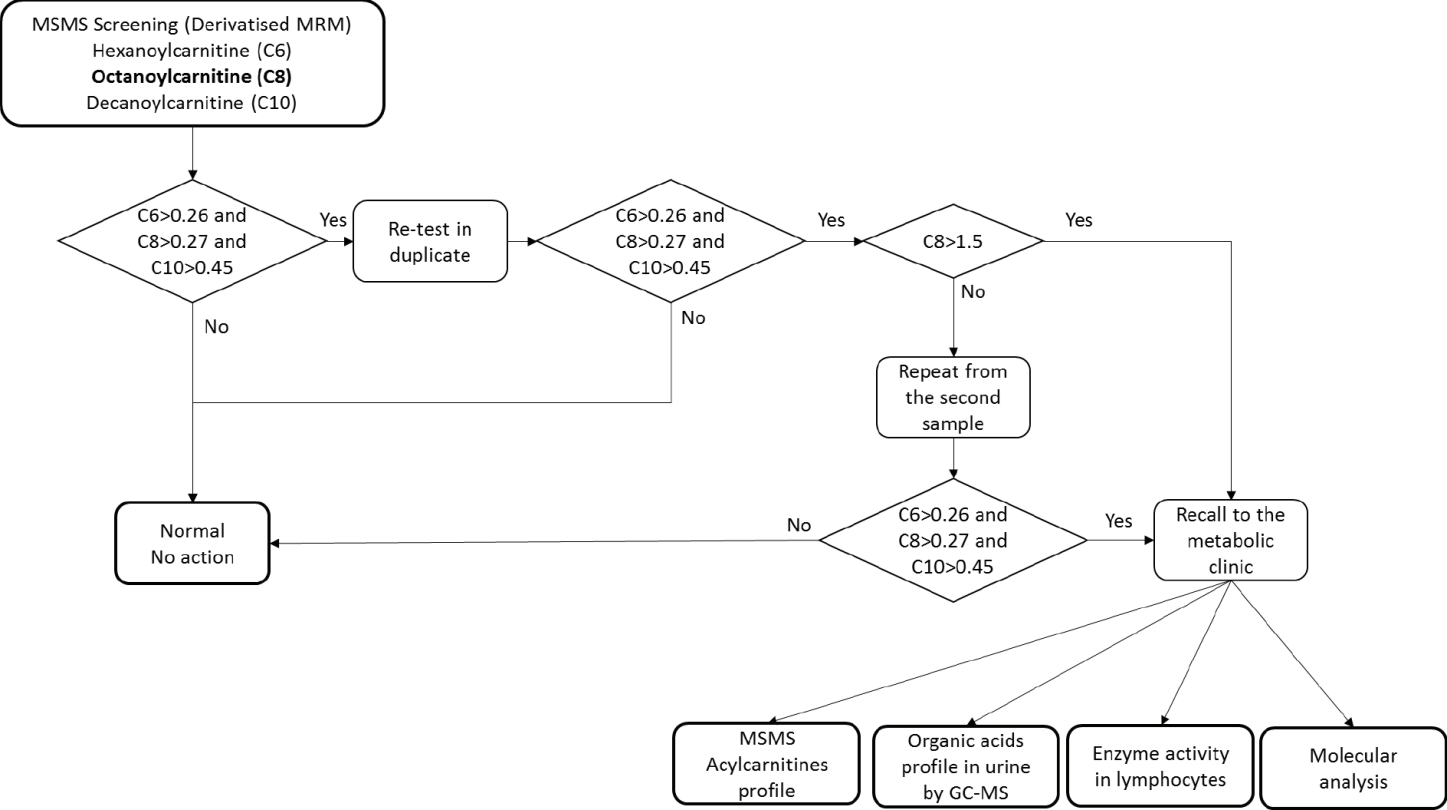
Screening protocol for MCAD deficiency. C6, C8, C10 concentrations were determined and expressed in μmol/L, MSMS - tandem mass spectrometry, MRM - Multiple Reaction Monitoring, GC-MS - Gas Chromatography-Mass Spectrometry.

The profile of the organic acids was measured using a gas chromatograph with a mass spectrometer (GCMS-QP2010 Ultra, SHIMADZU, Japan). The method was based on prior derivatisation with trimethylsilyl chloride. The pathognomonic profile included organic acids typical for MCAD deficiency, such as 7-hydroxyoctanoic acid, 5-hydroxyhexanoic, hexanoylglycine, and suberylglycine (Fig. 1).

Informed consent was obtained from all subjects involved in the study, as an obligatory rule of the newborn screening programme in Poland. The study was conducted in accordance with the Declaration of Helsinki for Human Research.

Statistical analysis was performed using Statistica 6.0 (StatSoft Inc.) software. Variable distributions were evaluated with the Kolmogorov-Smirnov test and descriptive statistics (means, standard deviations, medians and interquartile ranges) were calculated.

## 3. Results and Discussion

Since 2014, all newborns in Poland have been included in a screening programme for inborn errors of metabolism using tandem mass spectrometry (MS/MS). This test is performed at the Department of Screening and Metabolic Diagnostics at the Institute of Mother and Child (IMiD). To date, 3,806,166 newborns have been screened, with 94 cases of MCAD deficiency detected and confirmed by enzyme and/or molecular testing.

Analysis of the results from these newborns revealed in early infancy a consistent pattern in the dynamics and concentrations of acylcarnitines (C6, C8, C10) and the C8/C10 ratio, which are key diagnostic biomarkers for this condition in MS/MS analysis. However, one confirmed case of MCAD deficiency had a distinctly different biochemical profile.

Prior to MCAD deficiency being included in the screening panel, children with this disease were usually diagnosed between three and 24 months of age, typically following their first clinical episode [9]. In countries where MCAD deficiency is not part of routine screening, patients are still diagnosed only after the onset of symptoms. [11]. There are reports of metabolic crisis ending in death in a child with MCAD deficiency even before screening test results were available [6, 13, 14]. This emphasises the importance of investigating the factors that may cause delays in diagnosing this disorder.

In typical cases of MCAD deficiency, on the third and fourth days of life, very high concentrations of C8 (the primary biomarker) are observed along with elevated levels of C6 and C10, as well as a high C8/C10 ratio. After a few days, the biomarker levels decreased, but the C8 levels remained significantly above the reference values (Table 1). This supports the recommendation of collecting screening samples between the 36th and 72nd hours of life.

**Table 1.** C8 levels for typical MCAD cases from our cohort in newborn screening (first and second dried blood spot samples).

**Sample collection day of life**	**Number of cases**	**Reference range ≤1 month (µmol/L)**	**Mean (µmol/L)**	**SD (µmol/L)**	**Median (µmol/L)**	**Q1 (µmol/L)**	**Q3 (µmol/L)**
3	61	≤ 0.27	7.93	5.77	6.28	3.00	12.06
4	23		6.06	4.11	4.31	3.34	7.32
5	3		2.21	0.50	2.15	1.95	2.44
8	12		1.63	0.63	1.58	1.27	1.84
9	8		1.63	0.49	1.61	1.49	1.86
10	5		1.88	0.60	1.53	1.50	2.30
11	12		1.74	1.21	1.61	1.14	2.16
12	12		1.29	0.95	1.46	0.37	1.85

Data were presented as mean ± standard deviation and median (interquartile range, 1st–3rd quartile).

Relatively frequent false-positive results for C8, C6, and C10 have been reported, often due to factors such as low birth weight, significant weight loss after birth, parenteral nutrition, use of essential oils, or administration of medium-chain triglycerides (MCT) [10, 15]. Additionally, slight elevations in C8 concentrations have been documented in the blood samples collected from non-MCAD-deficient neonates on the first day of life. However, subsequent analysis of new blood samples revealed that these elevated levels had normalised.

It is established that a healthy newborn initially relies on glucose for energy. On the second to third days of life, glucose levels physiologically decline, triggering a rapid ketogenic response as part of extrauterine adaptation. During this process, energy is derived from ketone bodies, specifically 3-β-hydroxybutyrate (3-β-OHB) and acetoacetate (AcAc), which are synthesised in the liver from fatty acids [16].

If the activity of enzymes involved in fatty acid β-oxidation (FAO) is significantly impaired, hypoketotic hypoglycaemia may occur as a potentially life-threatening condition. This process results in the accumulation of medium-chain acyl-CoA, which conjugates with glycine to form hexanoylglycine and suberylglycine. These metabolites are excreted in urine and serve as diagnostic biomarkers for symptomatic MCAD deficiency in organic acid profiles analysed by gas chromatography–mass spectrometry (GC-MS) [1, 2, 10]. It is important to note that the urinary organic acid profile may appear normal during periods of metabolic stability.

As previously described, one case in the study group showed a profile that was significantly different from the others. The male neonate was delivered at 39 weeks of gestation via caesarean section because of suspected foetal asphyxia. He presented with severe perinatal asphyxia, with Apgar scores of 4, 6, and 7 at 1, 3, and 5 minutes, respectively. The umbilical cord blood pH was low at 6.7. On the first day of life, the neonate exhibited signs of encephalopathy, including lethargy, absence of Moro and sucking reflexes, and required respiratory support. Additionally, multiple episodes of myoclonic activity were observed. Based on the clinical assessment, the infant met the criteria for therapeutic hypothermia, which was initiated at six hours of life and maintained for 72 hours.

Due to the clinical condition and the need for treatment in the Neonatal Intensive Care Unit (NICU), the screening sample was collected only on the fifth day of life. The screening test revealed a slightly elevated C8 level (0.78 μmol/L; reference ≤0.27 μmol/L) and an increased C8/C10 ratio (7.15; reference ≤3.10), which, in accordance with the screening protocol, required a repeat sample.

The second dried blood spot sample, obtained only on the 34th day of life, demonstrated a marked elevation in the concentrations of C6 and C8 acylcarnitines, along with a significantly increased C8/C10 ratio. These findings provided strong biochemical evidence supporting the suspicion of MCAD deficiency (Table 2). On the 52nd day of life, the infant was admitted to the Department of Inborn Errors of Metabolism for further diagnostic evaluation and to establish a treatment plan. By the time the patient was called to the clinic, the child had not received MCT supplementation and his condition was stable.

**Table 2.** Results of the newborn with an atypical MCAD acylcarnitine profile.

**Acylcarnitne**	**MS/MS test**				

	**5 day**	**Reference range ≤1 month**	**34 day**	**52 day**	**Reference range >1 month**
C6 (µmol/L)	0.16	≤ 0.26	0.89	0.77	≤ 0.42
C8 (µmol/L)	0.78	≤ 0.27	5.61	3.30	≤ 0.68
C10 (µmol/L)	0.11	≤ 0.45	0.34	0.28	≤ 1.20
C8/C10 (ratio)	7.15	≤ 3.10	16.60	11.80	≤ 2.76

C6 – hexanoylcarnitine; C8 – octanoylcarnitine; C10 – decanoylcarnitine; C8/C10 – octanoylcarnitine/decanoylcarnitine ratio.

The diagnosis of MCAD deficiency was confirmed. Enzymatic activity testing in lymphocytes revealed residual activity of 3.4% (normal >35%). Molecular analysis identified two pathogenic variants in the *ACADM* gene: a missense variant NM_000016.5:c.985A>G (p.Lys329Glu) and a novel splicing variant NC_000001.11(NM_000016.5):c.118+1 G>A (p.?). On the 52nd day of life, the organic acid profile in urine (GC-MS) demonstrated the presence of MCAD deficiency specific metabolites, including slightly elevated levels of 7-hydroxyoctanoic acid, suberic acid, sebacic acid, and 2-hydroxysebacic acid, as well as suberylglycine and hexanoylglycine.

**Figure 2.**
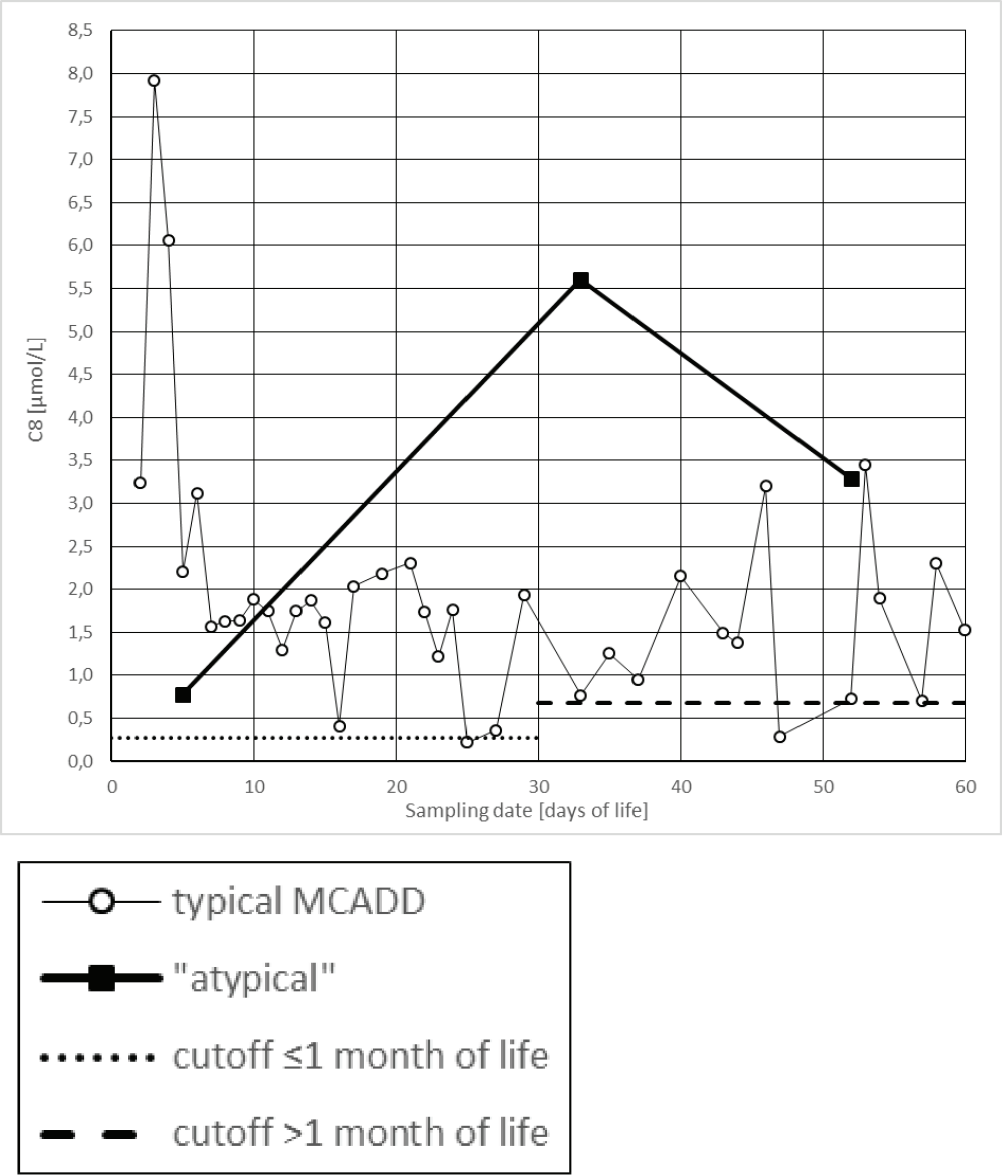
Octanoylcarnitine (C8) concentrations in typical MCAD cases (group mean) versus the atypical case, plotted by the day of life at sampling.

In this patient, an inverse trend in the concentration of C8 acylcarnitine was observed when compared with other neonates and infants diagnosed with MCAD deficiency (Fig. 2). While most MCAD deficiency cases exhibit a marked elevation in C8 levels within the first days of life, the initial newborn screening performed on day five in this patient showed only a slight increase above the upper reference limit. Some authors have noted that false-positive results are usually associated with such minimal elevations in C8, which raises concerns about the diagnostic utility of repeat testing in MCAD screening [17]. A significant rise in C8 concentration was detected on day 34 of life, deviating from the typical early postnatal peak characteristic of MCAD deficiency. Despite the blood sample being collected on the fifth day of life (later than the recommended collection time in Poland, which is on the third or fourth day), this minor delay is unlikely to account for the notably attenuated initial C8 concentration. In other countries, such as the United Kingdom, newborn screening is a standard procedure conducted between the fifth and eighth day of life. C8 is the primary biomarker for MCAD deficiency diagnosis in these countries [18, 19].

It is also important to emphasise that the patient received intravenous 10% glucose on the first day of life, which is a standard practice during the transport and treatment of critically ill newborns. Glucose administration has been demonstrated to inhibit ketogenesis, which consequently results in the normalisation or significant reduction of MCAD deficiency biomarker levels. It can thus be deduced that the intravenous administration of glucose therapy, initiated on the first day of life, was instrumental in the prevention of early metabolic decompensation. In addition, within the context of postnatal adaptations in lipid metabolism, glucose infusion has been hypothesised to modulate fatty acid oxidation, resulting in the initial suppression of C6, C8, and C10 concentrations. This biochemical response likely contributes to the delayed diagnosis.

## 4. Conclusions

The use of tandem mass spectrometry has enabled the inclusion of MCAD deficiency in newborn screening programmes. This case illustrates the diagnostic challenges that may arise, particularly in critically ill neonates receiving intensive care. The acylcarnitine profile that deviated from the typical pattern in this child was most likely due to the metabolic compensation induced by early intravenous glucose administration. This could have transiently inhibited ketogenesis and accumulation of diagnostic acylcarnitines (C6, C8, C10). In some cases, this may lead to false-negative results in newborn screenings. It is noteworthy that the implementation of a standardised screening protocol, specifically the recommendation to repeat blood sampling in cases of slightly elevated C8 concentrations, enabled timely diagnosis of the disorder. This highlights the importance of adhering to follow-up procedures, even when initial findings are inconclusive.

Furthermore, this case emphasises the pivotal function of interdisciplinary collaboration between the clinical teams and screening laboratories. The accurate interpretation of screening outcomes is contingent not only on biochemical parameters but also on the availability of comprehensive clinical information, including administered therapies. Such cooperation is imperative to enhance diagnostic precision and optimise patient outcomes in the context of newborn screening for inborn errors of metabolism.
